# Family history does not influence stress or major coping styles in adults with neurofibromatosis type 1

**DOI:** 10.1002/jgc4.70052

**Published:** 2025-05-13

**Authors:** Mikaela Bradley, Ashley Cannon, Bryce Brown, Kelly Taylor, Paul Moots, Emily McQuillen

**Affiliations:** ^1^ Master of Genetic Counseling Program Vanderbilt University School of Medicine Nashville Tennessee USA; ^2^ InformedDNA Inc St. Petersburg Florida USA; ^3^ Department of Clinical and Diagnostic Sciences University of Alabama at Birmingham Birmingham Alabama USA; ^4^ Medical Genomics Laboratory University of Alabama at Birmingham Birmingham Alabama USA; ^5^ Vanderbilt‐Ingram Cancer Center Vanderbilt University Medical Center Nashville Tennessee USA; ^6^ Department of Neurology Vanderbilt University Medical Center Nashville Tennessee USA; ^7^ Clinical Genomics Laboratory Vanderbilt University Medical Center Nashville Tennessee USA; ^8^ Present address: Ambry Genetics Aliso Viejo California USA

**Keywords:** coping, family history, lived experience, neurofibromatosis type 1, psychosocial, stress

## Abstract

Neurofibromatosis type 1 (NF1) is a genetic condition that affects about 1 in 3000 individuals. Approximately 50% of individuals with NF1 have a family history of the condition. Individuals with NF1 experience variable symptoms that contribute to increased stress. This study investigated whether a family history of NF1 influences levels of stress and coping strategies in adults with NF1. Adults with NF1 who live in the United States and speak English were recruited through the Children's Tumor Foundation's (CTF) NF Registry, CTF's NF Clinic Network, and the Neurofibromatosis Network. Participants completed a survey about their personal and family history of NF1, the Perceived Stress Scale 10‐Item Version (PSS‐10), the Brief Coping Orientation to Problems Experienced Inventory (Brief‐COPE), short response questions, and demographics. Overall, 547 of 646 responses met analysis criteria. Participants with affected parents were assigned to the inherited NF1 group (*n* = 222) and those with unaffected parents were assigned to the sporadic NF1 group (*n* = 325). No differences were found in mean PSS‐10 scores between the two study groups (*p* = 0.568). Females had significantly higher PSS‐10 scores than males (*p* < 0.001). After Bonferroni correction, no differences were found across Brief‐COPE subscales or major coping styles between the two groups. A stagewise multivariable regression indicated that 42% of the variance in PSS‐10 scores was accounted for by sex assigned at birth, age, problem‐focused, and avoidant coping styles (*R* ^2^ = 0.42, *p* < 0.001). Family history did not predict PSS‐10 scores alone or as an interaction variable with major coping styles. This study showed no significant differences in stress or major coping styles between adults with inherited versus sporadic NF1. However, other factors may influence the stress and coping experiences of adults with NF1. Fostering discussions about patients' stressors and coping strategies could help promote stress management.

**What is known about this topic?**

Due to the varied signs and severity of NF1, this condition is associated with significant uncertainty. Existing research has demonstrated that individuals with NF1 experience higher levels of stress compared to the general population.

**What does this paper add to the topic?**

This study examines whether the presence or absence of family history plays a role in the stress and coping experiences of adults with NF1. This is an important area of research to explore since approximately 50% of cases are inherited and 50% are *de novo*.

## 1 INTRODUCTION

Neurofibromatosis type 1 (NF1) is a genetic condition that affects approximately 1 in 3000 individuals (Radtke et al., 2020). NF1 is inherited in an autosomal dominant pattern, meaning that each child has a 50% chance of inheriting the condition from an affected parent. Approximately 50% of cases are inherited and 50% are *de novo* (Cimino & Gutmann, 2018). NF1 is thought to be fully penetrant and has wide inter‐ and intra‐family phenotypic variability (Rasmussen & Friedman, 2000). Common signs include but are not limited to café‐au‐lait macules, inguinal freckling, optic glioma, Lisch nodules, neurofibromas, long bone dysplasia, learning differences, malignant peripheral nerve sheath tumors (MPNSTs), and increased cancer risk (Cimino & Gutmann, 2018; Ly & Blakeley, 2019). Due to the varied signs and severity, NF1 is associated with significant uncertainty (Carrieri et al., 2016). This can impact an individual's surveillance, family planning, and mental health. Existing research has demonstrated that individuals with NF1 experience higher levels of stress compared to the general population and other cohorts as assessed by the PSS (Fishbein et al., 2022; Wang et al., 2012).

These studies provide a basis for further exploration of which factors contribute to increased stress within the NF1 population in order to identify potential areas to target during counseling. When studying stress, it is crucial to consider the role of coping. Coping is a dynamic process that modifies someone's stress experience. Adaptive coping strategies help mitigate stress, whereas maladaptive strategies can prolong the stressor. Typically, problem‐focused and emotion‐focused coping are considered to be adaptive. Lazarus and Folkman posit that problem‐focused coping aims to directly manage the stressor, while emotion‐focused coping focuses on regulating reactions to the stressor (Biggs et al., 2017). Meanwhile, maladaptive strategies that are used to disengage from the stressor are a form of avoidant coping. Coping styles are not mutually exclusive, and how individuals with NF1 cope may depend on their current stressors. Existing NF1 research about stress and coping provides important background information for this study. First, visible manifestations of NF1 can be a source of stress that leads to lower quality of life (QoL). Hamoy‐Jimenez et al. (2022) found that individuals with NF1 had significantly lower scores than the general population in six of eight domains of the SF‐36 QoL scale, which is a generic QoL measure. Similarly, a study exploring the impact of NF1 on well‐being found that cosmetic appearance affected self‐confidence across genders and all levels of NF1 severity and visibility (Crawford et al., 2015). Another study found that participants with NF1 expressed difficulties with coping and self‐acceptance, sharing that NF1 limited their coping skills due to depression, fatigue, and anticipatory stress (Rietman et al., 2018). Additionally, Bottesi et al. (2020) utilized the Coping Orientation to Problem Experiences (COPE) scale in NF1 patients and found that the “avoidance strategies” subscale was significantly correlated with physical distress and symptoms in individuals with more cutaneous neurofibromas. This highlights the role that visibility and symptom burden may have on individuals' experiences with NF1.

Evidence suggests that the inheritance pattern, uncertainty, and family structure are important considerations when studying stress and coping in adults with NF1. Carrieri et al. (2016) explored how family dynamics may impact people's experiences with NF1. One theme was variability in discourse about NF1 between family members. Some participants reported limiting their discussions about NF1 as a result of being well‐adjusted to their diagnosis, while others downplayed the impact of NF1 due to tense family dynamics. This suggests that family interactions may influence individuals' experiences with NF1. Similarly, Aghaei et al. (2023) interviewed individuals with NF1 and care providers and found that family interactions shaped individuals' social lives and mental health. Family interactions can have a positive or negative impact on these factors for individuals with NF1. Highlighting the theme of uncertainty, Crawford et al. (2015) reported participant concern about what NF1 symptoms their children would develop. Overall, these studies provide evidence that family experiences may impact how individuals navigate their NF1 diagnosis.

Although existing literature explores stress and coping in the overall NF1 population, it is not known if family history mediates these experiences in adults with NF1. This is a critical area of study as approximately 50% of NF1 cases are inherited and 50% are *de novo*. This study aims to investigate whether a reported family history of NF1 influences levels of stress and coping strategies in adults with NF1. First, it was hypothesized that individuals with inherited NF1 would experience lower stress compared to those with sporadic NF1. Being the first person in your family to experience a chronic illness can be unexpected, leading to increased stress (Iovino et al., 2023). Furthermore, the stress of a new diagnosis may be more salient in individuals with sporadic compared to inherited NF1. Second, it was hypothesized that individuals with inherited NF1 would have higher adaptive coping compared to those with sporadic NF1. Carrieri et al. (2016) found that having relatives who have experienced NF1 may expose patients to positive coping strategies they can adopt. Additionally, participants expressed that comparing their signs to those of family members helped them evaluate how NF1 may impact them in the future. Given the existing research about how family dynamics affect other aspects of this diagnosis, knowledge about the role of family history in stress and coping processes in adults with NF1 could help inform counseling approaches. For example, if individuals with sporadic NF1 are found to have higher stress or poorer adaptive coping, providers could screen patients by family history to identify individuals who may benefit from additional counseling.

## 2 MATERIALS AND METHODS

### 2.1 Overview

This cross‐sectional study was conducted using an online survey delivered via REDCap hosted at Vanderbilt University (Harris et al., 2009, 2019). REDCap is a secure web platform for creating and managing online surveys. This study was determined to be exempt by the Vanderbilt Institutional Review Board (#231166).

### 2.2 Participants and recruitment

Individuals were eligible to participate if they had a personal clinical and/or molecular diagnosis of NF1, were at least 18 years old, lived in the United States, and spoke English. Participation was limited to English‐speaking individuals living in the United States due to time and resource limitations. Participants were recruited through three methods: (1) the Children's Tumor Foundation's (CTF) NF Registry, (2) CTF's NF Clinic Network, and (3) the Neurofibromatosis Network. An email invitation was sent to 4,641 individuals on the NF Registry who met inclusion criteria and to 69 clinics within the NF Clinic Network. The Neurofibromatosis Network shared information about the study in their September 2023 Newsletter (distribution size was not available), via Facebook and Instagram posts on October 11, 2023, and linked it on their website for the duration of the study. Data were collected from September 1 to October 23, 2023. A response rate could not be calculated since this survey was distributed through multiple avenues. All participants who completed the survey had the option to receive a $10 Amazon e‐gift card, which were distributed on December 1, 2023.

### 2.3 Instrumentation

A 56‐question, anonymous survey was developed that included six components: (1) questions regarding their personal NF1 diagnosis, (2) questions about family members' NF1 status, (3) PSS‐10 scale items, (4) Brief‐COPE scale items (see scale descriptions under *Measures*), (5) short answer questions, and (6) demographics. Participants self‐reported perceived severity on a 4‐point scale (not, somewhat, moderate, and very severe). This scale was adapted from the Severity of Illness Index (Horn & Horn, 1986). A copy of the survey is available in the Data S1. Pictures and patient‐friendly language were used in addition to medical terms when discussing NF1 features. REDCap accessibility features, including increased text size and text‐to‐audio options, were enabled. The survey was reviewed by specialized providers and individuals with NF1 to gather feedback prior to distribution. This included a physician and three genetic counselors who have worked with NF1 patients and/or previously conducted NF1 research. Participants read an information sheet and completed eligibility questions before beginning the survey.

#### 2.3.1 Measures

Perceived Stress Scale 10‐Item Version (PSS‐10): The PSS‐10 is a self‐report measure that assesses stress in the past month on a scale from 1 (never) to 4 (very often) (Cohen et al., 1983). Higher scores indicate higher perceived stress (range: 1–40; Cohen & Williamson, 1988). The PSS‐10 has been utilized in studies with individuals who have NF1, which allows for comparison to the current study and shows validity in the target population (Fishbein et al., 2022; Wang et al., 2012). This measure has also been used to study stress in other chronic diseases (Downing et al., 2012; Luc et al., 2023; Sun et al., 2019). Internal consistency in this sample was good (*α* = 0.89; Cronbach, 1951).

Brief Coping Orientation to Problems Experienced Inventory (Brief‐COPE): The Brief‐COPE is a 28‐item self‐report questionnaire that assesses coping reactions through 14 two‐item subscales: self‐distraction, active coping, denial, substance use, emotional support, instrumental support, behavioral disengagement, venting, positive reframing, planning, humor, acceptance, religion, and self‐blame (Carver, 1997; Carver et al., 1989). Coping is assessed on a scale from 1 (not at all) to 4 (a lot), with higher scores indicating more frequent use of the coping trait. Scores for each subscale range from 2 to 8. The full COPE scale has been used in at least one study with individuals who have NF1, and the Brief‐COPE has been used in healthcare settings (Bottesi et al., 2020; Eisenberg et al., 2012; Halcomb et al., 2022). The subscales were grouped into emotion‐focused, problem‐focused, and avoidant major coping styles, which was consistent with past studies (Khoury et al., 2021; Schnider et al., 2007). Overall internal consistency in this sample was good (*α* = 0.86; Cronbach, 1951). Alpha coefficients for subscales are listed in Table 5.

The PSS‐10 and Brief‐COPE have previously been used together in studies that measured stress and coping (Awoke et al., 2021; Furman et al., 2018; Khoury et al., 2021; Luc et al., 2023).

#### 2.3.2 Assigning study groups

Participants were assigned to the “inherited NF1” or “sporadic NF1” group based on their response to the question, “Has one of your biological parents been diagnosed with NF1?” Participants who responded “yes” were assigned to the “inherited NF1” group, and participants who responded “no” were assigned to the “sporadic NF1” group. Due to the variability of family dynamics, the health status of relatives is not always known. Therefore, this study used the phrase “inherited NF1” to refer to cases with a *reported* family history. Participants with NF1 with unaffected parents were classified as sporadic NF1, even if they had children with NF1. Individuals who reported that they were adopted or did not know their family history were excluded from analysis (*n* = 45).

### 2.4 Data analysis

Quantitative analysis was performed in SPSS Version 29.0.0 (IBM Corp., 2022). Frequency and descriptive statistics were computed for demographics and NF1‐related characteristics. Total PSS‐10 scores and Brief‐COPE subscale scores were calculated as outlined in the literature (Carver, 1997; Cohen et al., 1983). Independent *t*‐tests and Mann–Whitney *U* tests were used to compare PSS‐10 and Brief‐COPE scores between groups. Normality was assessed by histogram distribution and skewness and kurtosis (Kim, 2013; Mishra et al., 2019). Spearman's rho correlation was used to explore the relationship between PSS‐10 and Brief‐COPE scores as well as additional variables. A stagewise multivariable linear regression assessed the influence of modifying variables on stress. Variables were manually selected based on demographics and NF1 characteristics that were correlated with PSS‐10 scores (Table 7). A stagewise approach was selected in an attempt to control for additional factors (e.g., demographics) that may influence the model before adding coping strategies, family history, and their interaction variables. A nominal *p*‐value threshold of *p* < 0.05 was used to assume statistical significance. Bonferroni correction was used to adjust for multiple analyses in hypothesis testing and is noted when reporting results.

## 3 RESULTS

### 3.1 Demographics

A total of 749 participants completed the eligibility screening and began the survey, of which 646 participants finished the survey and met inclusion criteria (86.2% completion rate). Fifty‐four responses were removed due to duplicate submissions or erroneous data entry. Additionally, responses from participants who were adopted or had unknown parental NF1 status were excluded from analysis due to small sample sizes (*n* = 45). Overall, 547 responses were used for analysis, of which 222 (40.6%) reported having an affected parent(s) and 325 (59.4%) reported having unaffected parents. Most participants were female (*n* = 406, 74.2%) and non‐Hispanic/Latino white (*n* = 434, 79.3%) with an average current age of 44.34 years and more than a high school degree (*n* = 413, 75.8%). Differences in demographic breakdown between the two study groups are noted in Table 1.

**TABLE 1:** Participant demographics.

	Inherited (*n* = 222)	Sporadic (*n* = 325)	All participants (*n* = 547)	Differences in demographic breakdown^a^
Current age				
Average (range)	45.87 (18–87)	43.69 (18–83)	44.34 (18–87)	*p* = 0.025
Age of diagnosis	***n* (%)**	***n* (%)**	***n* (%)**	
0–2 years (infancy)	94 (42.3)	119 (36.6)	213 (38.9)	
3–6 years (early childhood)	38 (17.1)	67 (20.6)	105 (19.2)	
7–12 years (late childhood)	31 (14.0)	44 (13.5)	75 (13.7)	
13–17 years (adolescence)	14 (6.3)	26 (8)	40 (7.3)	
18 years or older (adults)	35 (15.8)	65 (20)	100 (18.3)	
Unknown	10 (4.5)	4 (1.2)	14 (2.6)	
Sex assigned at birth (SAAB)				
Female	172 (77.5)	234 (72)	406 (74.2)	
Male	50 (22.5)	91 (28)	141 (35.8)	
Race^b^				
American Indian/Alaska Native/Native American	6 (2.7)	8 (2.5)	14 (2.6)	
Asian/Asian American	1 (0.5)	15 (4.6)	16 (2.9)	*p* = 0.004
Black/African American	12 (5.4)	11 (3.4)	23 (4.2)	
Middle Eastern/North African	1 (0.5)	3 (0.9)	4 (0.7)	
Native Hawaiian/Pacific Islander	–	3 (0.9)	3 (0.5)	
White/Caucasian	207 (93.2)	284 (87.4)	491 (89.8)	*p* = 0.026
Other/prefer not to say	3 (1.4)	17 (5.2)	20 (3.6)	*p* = 0.033
Ethnicity				
Hispanic or Latine	19 (8.6)	29 (8.9)	48 (8.8)	
Non‐Hispanic or Latine	194 (87.4)	276 (84.9)	470 (85.9)	
Prefer not to say	9 (4.1)	20 (6.2)	29 (5.3)	
Education				
Less than a high school degree	7 (3.2)	16 (4.9)	23 (4.2)	
High school degree or equivalent	57 (25.7)	52 (16)	109 (19.9)	*p* = 0.005
Some college, no degree	43 (19.4)	56 (17.2)	99 (18.1)	
Trade or tech school	10 (4.5)	15 (4.6)	25 (4.6)	
Associate's degree	32 (14.4)	34 (10.5)	66 (12.1)	
Bachelor's degree	48 (21.6)	82 (25.2)	130 (23.8)	
Master's degree	20 (9)	56 (17.2)	76 (13.9)	*p* = 0.006
Doctorate degree	5 (2.3)	12 (3.7)	17 (3.1)	
Other	–	2 (0.6)	2 (0.4)	
Employment				
Full‐time employment	118 (53.2)	145 (44.6)	263 (48.1)	
Part‐time employment	21 (9.5)	41 (12.6)	62 (11.3)	
Not currently employed	16 (7.2)	27 (8.3)	43 (7.9)	
Disability	31 (14)	44 (13.5)	75 (13.7)	
Retired	20 (9)	43 (13.2)	63 (11.5)	
Student	5 (2.3)	12 (3.7)	17 (3.1)	
Other/prefer not to say	11 (5)	13 (4)	24 (4.4)	

^a^ Differences in demographic breakdown between the inherited and sporadic groups were assessed by Chi‐Square, Fisher's Exact, or Mann–Whitney *U* tests depending on data type and sample size.

^b^ Participants could select multiple responses for race, resulting in totals over 100%.

Additional information about participants' NF1 diagnosis, including perceived severity and affected family members is reported in Table 2. The distribution of perceived severity was the same between study groups (*X* ^2^ = 6.55, *p* = 0.088). There was an equal distribution of individuals who inherited NF1 from their mother versus their father. The difference in the distribution of other affected relatives between study groups is expected, given that we would not anticipate many individuals with sporadic NF1 to have affected relatives apart from biological children or by chance.

**TABLE 2:** Additional participant characteristics.

	Inherited NF1	Sporadic NF1
Number of NF1 features^a^	**M ± SD (range)**	**M ± SD (range)**
	5.48 ± 2.01 (1–13)	5.35 ± 1.93 (1–13)
Perceived severity	***n* (%)**	***n* (%)**
Not severe	55 (24.8)	101 (31.1)
Somewhat severe	79 (35.6)	126 (38.8)
Moderately severe	69 (31.1)	82 (25.2)
Very severe	19 (8.6)	16 (4.2)
Affected parent		
Mother	114 (46.8)	–
Father	104 (51.4)	–
Both	1 (0.5)	–
Other affected relatives		
Yes	177 (79.7)	87 (26.8)
No	45 (20.3)	238 (73.2)

^a^ Table 3 contains a list of NF1‐related features that participants could select and their frequencies.

NF1‐related features reported by participants were also assessed (see Table 3). Participants selected from a list of well‐known features associated with NF1 and could write in additional features (Cimino & Gutmann, 2018; Friedman, 1998). The current study population had phenotypic variability.

**TABLE 3:** Frequency of NF1 features.

	Inherited (*n* = 222)	Sporadic (*n* = 325)
Café au lait spots	87.4%	91.7%
Cutaneous neurofibromas	84.7%	80.3%
Freckling	76.1%	78.8%
Lisch nodules	58.6%	56%
Plexiform neurofibromas	60.8%	51.7%
Optic glioma	17.6%	20.0%
Learning differences	53.6%	51.4%
Long bone dysplasia	10.4%	6.2%
Scoliosis	39.2%	40.9%
MPNST	3.6%	3.7%
Seizures	7.7%	6.2%
Osteoporosis	16.2%	14.8%
Hypertension	25.2%	26.5%
Additional symptoms	6.8%	7.4%

### 3.2 PSS‐10 scores between participants with inherited NF1 and sporadic NF1

Total PSS‐10 scores were computed with reverse scoring of items 4, 5, 7, and 8 since they are positively worded statements (Cohen et al., 1983). Independent *t*‐tests were used to compare mean PSS‐10 scores between the two study groups (see Table 4). There was no statistically significant difference in average PSS‐10 scores between participants with inherited NF1 (19.68 ± 7.62) and sporadic NF1 (19.30 ± 7.72; *p* = 0.568). Overall, females (20.37 ± 7.15) had significantly higher mean PSS‐10 scores compared to males (16.82 ± 8.50; *p* < 0.001). To further assess the influence of family history on stress scores, we stratified participants by sex assigned at birth (SAAB) and parental NF1 status. No significant differences were identified between females with inherited NF1 and females with sporadic NF1 (*p* = 0.753) or between males with inherited NF1 and males with sporadic NF1 (*p* = 0.964).

**TABLE 4:** PSS‐10 scores by family history and sex assigned at birth.

	Mean ± SD	Range	*t*	*p*
Inherited NF1	19.68 ± 7.62	0–38	0.57	0.568
Sporadic NF1	19.30 ± 7.72	0–39		
Females	20.37 ± 7.15	0–38	4.45	<0.001^a^
Males	16.82 ± 8.50	0–39		
Females with inherited NF1	20.50 ± 7.19	1–38	−0.32	0.753
Females with sporadic NF1	20.27 ± 7.13	0–36		
Males with inherited NF1	16.86 ± 8.41	0–35	−0.05	0.964
Males with sporadic NF1	16.79 ± 8.59	0–39		

^a^ Bonferroni correction: *p* < 0.0125 (0.05/4).

### 3.3 Brief‐COPE scores between participants with inherited NF1 and sporadic NF1

Median Brief‐COPE subscale scores were calculated since no total coping score is generated from this measure (see Table 5). The subscales were condensed into three groups to represent the major coping styles: emotion‐focused, problem‐focused, and avoidant coping. Mann–Whitney *U* tests were used since normality was not met for all subscales. Distributions of scores for inherited and sporadic NF1 were similar for all subscales except for positive reframing. Mean rank is used when the distribution is not similar and the median cannot be reported. Participants with inherited NF1 (mean rank: 291.7) scored higher on the positive reframing subscale compared to those with sporadic NF1 (mean rank: 261.9, *U* = 32,151.5, *p* = 0.028). However, this was no longer statistically significant when a Bonferroni correction was applied with a new significance level of *p* < 0.0029. No statistically significant differences were identified across other Brief‐COPE subscales or major coping styles.

**TABLE 5:** Brief‐COPE scores for participants with inherited and sporadic NF1.

	Median (mean rank)		*U*	*p*	*α*
	Inherited NF1	Sporadic NF1			
Brief‐COPE subscales					
Acceptance	6 (279.19)	6 (270.45)	34,972.0	0.517	0.65
Active coping	5 (271.14)	5 (275.96)	36,711.0	0.722	0.72
Behavioral disengagement	3 (277.06)	3 (271.91)	35,396.5	0.692	0.73
Denial	2 (275.41)	2 (273.03)	35,761.0	0.844	0.74
Emotional support	5 (277.71)	4 (271.47)	35,251.5	0.645	0.83
Humor	4 (286.55)	3 (265.43)	33,288.5	0.112	0.84
Instrumental support	4 (279.78)	4 (270.05)	34,792.5	0.472	0.78
Planning	5 (278.71)	5 (270.78)	35,029.0	0.559	0.74
Positive reframing	(291.7)^a^	(261.9)^a^	32,151.5	0.028^b^	0.72
Religion	4 (288.00)	4 (264.43)	32,966.0	0.079	0.89
Self‐blame	4 (278.97)	4 (270.61)	34,972.0	0.527	0.76
Self‐distraction	5 (282.83)	5 (267.97)	34,115.5	0.273	0.53
Substance use	2 (271.24)	2 (275.88)	36,687.5	0.616	0.93
Venting	4 (271.03)	4 (276.03)	36,722.5	0.712	0.61
Major coping styles					
Emotion‐focused	27.5 (284.82)	27 (266.61)	33,673.5	0.185	0.73
Problem‐focused	19 (282.91)	19 (267.91)	34,096.0	0.275	0.85
Avoidant	14 (278.40)	13 (271.00)	35,099.0	0.589	0.69

^a^ The median was not reported because distributions of positive reframing scores are not similar per visual inspection. Therefore, mean rank values are used for comparison per the Mann–Whitney *U* Test.

^b^ Bonferroni correction *p* < 0.0029 (0.05/17).

### 3.4 Correlations between stress, coping strategies, and additional features

Spearman's rho correlation was used to investigate the relationship between Brief‐COPE subscales and PSS‐10 scores by study group, as seen in Table 6 (Akoglu, 2018). Behavioral disengagement, denial, and self‐blame had a moderate positive correlation with PSS‐10 scores for both groups. The remaining subscales had weak or no correlations with PSS‐10 scores. For both groups, avoidant coping had a moderate positive correlation, emotion‐focused coping had a weak positive correlation, and problem‐focused coping was not correlated with PSS‐10 scores.

**TABLE 6:** Correlations between Brief‐COPE subscales and PSS‐10 by NF1 parental status.

	Inherited NF1		Sporadic NF1	
	*r*_s_	Interpretation	*r*_s_	Interpretation
Brief‐COPE subscales				
Acceptance	−0.24	Weak negative	−0.26	Weak negative
Active coping	−0.01	No correlation	−0.14	Weak negative
Behavioral disengagement	0.57	Moderate positive	0.59	Moderate positive
Denial	0.47	Moderate positive	0.43	Moderate positive
Emotional support	−0.02	No correlation	−0.14	Weak negative
Humor	0.17	Weak positive	−0.03	No correlation
Instrumental support	0.14	Weak positive	−0.03	No correlation
Planning	0.12	Weak positive	0.09	No correlation
Positive reframing	−0.15	Weak negative	−0.26	Weak negative
Religion	0.02	No correlation	−0.14	Weak negative
Self‐blame	0.56	Moderate positive	0.58	Moderate positive
Self‐distraction	0.30	Weak positive	0.19	Weak positive
Substance use	0.16	Weak positive	0.22	Weak positive
Venting	0.30	Weak positive	0.32	Weak positive
Major coping types				
Emotion‐focused coping	0.24	Weak positive	0.13	Weak positive
Problem‐focused coping	0.04	No correlation	−0.09	No correlation
Avoidant coping	0.58	Moderate positive	0.57	Moderate positive

Additional bivariate analyses identified other factors that may influence PSS‐10 scores. Table 7 shows the correlations between participant characteristics and PSS‐10 scores for all respondents (Akoglu, 2018). Higher perceived severity of diagnosis, higher number of NF1 features, and presence of learning differences had weak positive correlations with PSS‐10 scores. Older current age, higher levels of education, and being male had weak negative correlations with PSS‐10 scores. No correlations were found between PSS‐10 scores and age of diagnosis, number of affected relatives, or race.

**TABLE 7:** Correlations between PSS‐10 scores and participant characteristics.

	*r*_s_	Interpretation
Sex assigned at birth (male/female)	−0.19	Weak negative
Current age	−0.18	Weak negative
Age of diagnosis	−0.03	No correlation
Level of education	−0.13	Weak negative
Race (white/non‐white)	−0.02	No correlation
Perceived severity	0.17	Weak positive
Number of affected relatives	0.04	No correlation
Number of NF1 features	0.13	Weak positive
Learning differences (yes/no)	0.25	Weak positive

### 3.5 Regression model

A stagewise multivariable linear regression was conducted to evaluate whether demographics, family history, major coping styles, and their interactions predicted PSS‐10 scores (see Table 8). Given the results of bivariate correlations, demographics and NF1 characteristics were included to control for modifying variables. The variables of race (white/non‐white), college degree (yes/no), and perceived severity (high (very and moderately severe)/low (somewhat and not severe)) were dichotomized for this analysis. The data met the assumptions of linearity, independence of residuals, homoscedasticity, collinearity, and normality.

**TABLE 8:** Stagewise multivariable regression (*n* = 544^a^).

Variables	Model 1			Model 2			Model 3		
	*B* (SE)	*t*	*p*	*B* (SE)	*t*	*p*	*B* (SE)	*t*	*p*
Constant	25.91 (1.41)	18.38	<0.001	23.22 (1.6)	14.54	<0.001	23.06 (1.4)	16.53	<0.001
Sex assigned at birth	−3.67 (0.73)	−5.05	<0.001^b^	−3.67 (0.71)	−5.15	<0.001^c^	−3.06 (0.59)	−5.19	<0.001^d^
Current age	−0.080 (0.02)	−3.75	<0.001^b^	−0.10 (0.02)	−4.69	<0.001^c^	−0.09 (0.02)	−4.95	<0.001^d^
Race	−1.34 (1.05	−1.28	0.201	−1.18 (1.03)	−1.15	0.252	−0.42 (0.86)	−0.48	0.630
College degree	−1.66 (0.65)	−2.57	0.011^b^	−1.02 (0.64)	−1.59	0.114	−0.16 (0.54)	−0.30	0.764
Number of symptoms				0.39 (0.16)	2.48	0.013	0.22 (0.13)	1.67	0.096
Perceived severity				3.10 (0.68)	4.55	<0.001^c^	1.32 (0.58)	2.28	0.023
Family history							−0.10 (0.53)	−0.19	0.848
Emotion coping							0.18 (0.09)	2.06	0.040
Problem coping							−0.35 (0.10)	−3.55	<0.001^d^
Avoidant coping							1.03 (0.12)	8.96	<0.001^d^
Family history × emotion coping							−0.08 (0.12)	−0.65	0.517
Family history × problem coping							−0.01 (0.13)	−0.08	0.937
Family history × avoidant coping							0.03 (0.15)	0.23	0.818

Model summary	Model 1	Model 2	Model 3
*R* ^2^/*F*	0.08/12.21	0.13/13.64	0.42/29.12
Δ*R* ^2^/Δ*F*	0.08/12.21	0.05/15.22	0.28/36.92
*p*	<0.001^b^	<0.001^c^	<0.001^d^

^a^ Two cases were excluded due to incomplete data, and one case was removed as an outlier.

^b^ Model 1 ‐ significant after Bonferroni correction *p* < 0.0125 (0.05/4).

^c^ Model 2 ‐ significant after Bonferroni correction *p* < 0.0083 (0.05/6).

^d^ Model 3 ‐ significant after Bonferroni correction *p* < 0.0038 (0.05/13).

Model 1 included the demographics of SAAB, current age, race, and having a college degree. This model was statistically significant, *R* ^2^ = 0.08, *F*(4,540) = 12.21, *p* < 0.001, and accounted for 8.3% of the variance in PSS‐10 scores. Model 2 included the addition of perceived severity and the number of NF1 features. This model was statistically significant, *R* ^2^ = 0.13, *F*(6,538) = 13.64, *p* < 0.001, and accounted for an additional 4.9% of the variance in PSS‐10 scores (Δ*R* ^2^ = 0.05, *p* < 0.001). Model 3 included the addition of family history, major coping styles, and their interaction variables. This model (full model) was statistically significant, *R* ^2^ = 0.42, *F*(13,531) = 29.12, *p* < 0.001; adjusted *R* ^2^ = 0.40, and accounted for an additional 28.4% of the variance in PSS‐10 scores (Δ*R* ^2^ = 0.28, *p* < 0.001). Family history was not a significant predictor when added to the model alone or as an interaction variable with the major coping styles. SAAB, current age, problem‐focused, and avoidant coping were significant predictors of stress scores in the final model when the Bonferroni correction was applied. Overall, the variables assessed accounted for 42% of the variance in PSS‐10 scores. Consequently, 58% of the variance in PSS‐10 scores was unaccounted for by factors in this model.

## 4 DISCUSSION

### 4.1 The influence of family history of NF1 on stress and coping

The first study aim was to explore whether family history influences the perceived stress levels in adults with NF1. The hypotheses that participants with inherited NF1 would (1) have lower PSS‐10 scores and (2) endorse higher use of adaptive coping strategies were not substantiated. The lack of differences in PSS‐10 scores between participants with inherited and sporadic NF1 suggests that other personal or medical factors may be drivers of stress in adults with NF1. Additionally, since the PSS‐10 assesses stress in the past month, scores could be different if individuals were in a period of high NF1‐related stress. The second aim explored whether family history influences the coping strategies and major coping styles used by adults with NF1. No differences were identified in the major styles of emotion‐focused, problem‐focused, or avoidant coping between the two study groups. When evaluating the Brief‐COPE subscales, a difference was observed on the measure of positive reframing. However, this finding was no longer significant when the Bonferroni correction was applied. Further research is needed to explore what factors may influence the use of different coping strategies and major coping styles in individuals with NF1.

Some evidence supports the current findings that there are no differences in stress and coping between participants with inherited and sporadic NF1. For example, Solem et al. (2020) found that unaffected parents had higher levels of NF1 knowledge than affected parents. This knowledge‐seeking from unaffected parents may result in increased communication about NF1 with their affected child. Consequently, this could lead to decreased stress or increased adaptive coping skills that are on par with those who have a family history of this condition. However, further studies are needed to investigate this hypothesis.

We further explored the relationship between stress, coping, and family NF1 status. Avoidant coping had a moderate positive correlation with PSS‐10 scores for both study groups. Specifically, the Brief‐COPE subscales of behavioral disengagement, denial, and self‐blame influenced this correlation. This is consistent with studies showing that avoidant or maladaptive coping is associated with higher stress levels (Zhang et al., 2022).

### 4.2 Additional variables that influence stress

Although family history was not found to be a predictor of stress, several variables were identified as modifiers of stress. First, the finding that females had significantly higher levels of stress compared to males is consistent with literature suggesting that females in the general population have higher stress than males when assessed by the PSS‐10 (Cohen & Janicki‐Deverts, 2012; Graves et al., 2021; Kneavel, 2021). Other studies have shown that females have higher perceived stress than males in conditions including melanoma, infective endocarditis, and Huntington disease (Downing et al., 2012; Malinauskas et al., 2022; Russell et al., 2018). Females also had higher perceived stress than males in people with a self‐reported higher body weight (Wijayatunga et al., 2023). This provides evidence that appearance may play a more prominent role in stress for females. The results of the current study differ from Wang et al. (2012), which did not find a significant difference in stress across genders in individuals with all types of NF using the PSS 4‐Item Version. This may be due to factors such as differences in sample size, sex distribution, and number of PSS items assessed. Notably, some studies evaluate stress through related measures such as depression or QoL. Hamoy‐Jimenez et al. (2022) found that women with NF1 had lower scores on the perceived physical appearance, anxiety, emotional health domains, and total scores of the PedsQL‐NF1 measure compared to men with NF1. Similar findings were reported by Cieza Rivera et al. (2024), who found that women had poorer QoL compared to men in an NF1 population. Additionally, Cohen et al. (2015) found that women with NF1 had more depressive symptoms compared to men with NF1. This supports our finding that females with NF1 may have higher levels of stress compared to males with NF1, regardless of family history. However, it is important to acknowledge that these results do not account for systemic factors. Males often underreport symptoms, and females may have heightened awareness of their appearance and associated stress due to societal beauty expectations (Rodgers et al., 2024; Smith et al., 2018).

Evaluation of other demographics yielded additional correlations between PSS‐10 scores. First, being older was loosely correlated with lower PSS‐10 scores. Research suggests that stress may decrease as we age because older individuals may perceive events as less stressful (Cohen & Janicki‐Deverts, 2012). Additionally, higher education was loosely correlated with lower PSS‐10 scores. Cieza Rivera et al. (2024) found that lower educational levels in NF1 patients were associated with poorer QoL scores. It is important to consider how targeted interventions could assist with the management of learning differences in the NF1 population, which could decrease some aspects of stress.

After looking at differences in stress by demographics, additional correlations between PSS‐10 scores and NF1 characteristics were explored. Using Spearman's rho correlation, higher perceived severity of diagnosis and a higher number of NF1 features were loosely correlated with higher PSS‐10 scores. This was expected since having a more severe diagnosis could increase the disease burden and is consistent with previous studies in the NF1 population (Fleming et al., 2023; Yamauchi & Suka, 2023). The correlation in this study may have been weak since severity is subjective and may be better assessed through a more thorough measure compared to self‐report.

In the overall regression model, SAAB, current age, problem‐focused coping, and avoidant coping were predictors of PSS‐10 scores when a Bonferroni correction was applied. Altogether, the model accounted for 42% of the variance in stress scores. However, this means that 58% of the variance in stress scores was unaccounted for by these variables. Other factors that could impact stress include but are not limited to, social support, access to care, non‐NF1‐related stressors, and personal characteristics such as NF1‐related anxiety disorder or other mental health concerns. This list of variables was generated after analysis based on short answer responses collected as a second part of this study. These variables were not directly incorporated into the survey, given that our study focus was evaluating the impact of family history on stress and coping. This highlights the need for further research that explores sources of stress in patients with NF1. Learning which factors are primary drivers of stress for individuals with NF1 can allow for the development of more targeted counseling interventions based on their personal characteristics. For example, results from the current study suggest that paying additional attention to stress levels in females with NF1 may be warranted. Future studies should assess what format of resources and interventions would be most beneficial to the NF1 population, such as additional handouts about stress management or support group opportunities.

### 4.3 Study limitations

Although we had a large sample, it was predominantly female and white. NF1 lacks a known biased sex ratio or founder effect in a specific ethnicity (Ly & Blakeley, 2019). Additionally, this study was limited to English‐speaking individuals in the United States, and about three‐fourths had more than a high school degree. As such, these results may not reflect the experiences of groups that were not well represented. Future research could aim to achieve a representative sample of the overall NF1 population to determine if the results would be different.

The study also had methodological limitations. First, the survey relied on self‐reported perceived severity and NF1 diagnoses. Some genetic conditions have phenotypic overlap with NF1 (e.g., Legius syndrome), and there was no clinical corroboration of participants' diagnoses. Second, the PSS‐10 measures stress over the past month and may not accurately reflect chronic stress.

Despite three recruitment sources, our study is subject to ascertainment bias since individuals involved in a registry may be more inclined to participate in research. Similarly, our study may lack individuals who are not in support groups or did not recently attend an NF clinic.

Lastly, approximately 50% of individuals with NF1 have learning differences, and they were reported by 52% of our study participants. Despite efforts to increase accessibility through lay language, multi‐modal functionality, and text‐to‐audio functions, some individuals may have been unable to participate.

### 4.4 Practice implications

The study results have practical implications for medical professionals, including geneticists, genetic counselors, and others caring for adults with NF1. Knowledge about what factors may influence the stress experienced by individuals with NF1 can inform providers about which patient subpopulations may be at risk for higher stress levels. Overall, the presence or absence of a family history of NF1 did not result in statistically significant differences in levels of stress or coping. This could be important for clinical genetic counselors to consider during sessions, as these results suggest that family history status does not influence stress levels or major coping styles. Therefore, counseling should focus on the individual stressors and experiences of patients with NF1.

### 4.5 Future research ideas

Future research could explore if family history impacts other aspects of patients' experiences with NF1, such as the time to diagnosis or health communication. Additionally, studies could explore how a family history of NF1 and parental discussions about NF1 may influence affected and unaffected children and adolescents. Other studies could further evaluate the role of perceived severity of NF1 and other qualitative factors on stress and coping through validated measures and clinical correlation.

## 5 CONCLUSIONS

Many factors may influence the stress and coping experience of adults with NF1. This study suggests that there are no statistically significant differences in overall stress levels and coping strategies between adults with inherited versus sporadic NF1. Factors such as SAAB, current age, perceived severity, and level of education were found to correlate with participants' stress experiences. Fostering discussions about a patient's primary stressors and helping them identify positive coping strategies could help promote stress management for patients with NF1.

## AUTHOR CONTRIBUTIONS

Author Mikaela Bradley confirms that she had full access to all the data and agrees to be accountable for all aspects of the work in ensuring that questions related to the accuracy or integrity of any part of the work are appropriately investigated and resolved. All authors take responsibility for the research and have each made significant contributions to the manuscript in concept, analysis, drafting, and revision. All the authors gave final approval for this version to be published.

## CONFLICT OF INTEREST STATEMENT

Emily McQuillen is employed by Ambry Genetics. Ashley Cannon is employed by InformedDNA. Mikaela Bradley, Bryce Brown, Kelly Taylor, and Paul Moots have no conflicts of interest to declare.

## ETHICS STATEMENT

Human Studies and Informed Consent: This study was granted an exemption by the Vanderbilt University IRB and conformed to standards stated in the Declaration of Helsinki. Formal informed consent was not required as data in this study was anonymous, and procedures were determined to be low‐risk. Participants read a study information sheet prior to beginning the online survey. Implied informed consent was obtained for individuals who elected to complete the survey.

Animal Studies: No non‐human animal studies were carried out by the authors for this article.

## Supporting information


Data S1.

## Data Availability

The data that support the findings of this study are available from the corresponding author upon reasonable request.
