# Supplementary material for: Family history does not influence stress or major coping styles in adults with neurofibromatosis type 1
Source: J Genet Couns. 2025 May 13;34(3):e70052. doi: 10.1002/jgc4.70052 (PMC12075914; doi:10.1002/jgc4.70052)
Supplement: Supplementary file 1 — Data S1. [file JGC4-34-0-s001.pdf]

The influence of a reported family history of NF1 on perceived stress and coping styles in adults with NF1

Thank you for your interest in our research study! Prior to beginning the survey, you will be asked to review the information sheet below that explains the purpose of the study, reviews risks, and discusses privacy.If you have any questions about this research study, please contact Mikaela Bradley at mikaela.r.bradley@vanderbilt.edu, or my advisor, Emily Solem at emilysolem@gmail.com

Research Study Information Sheet This survey has been distributed to the NF Registry, NF Clinic Network, and NF Network. If you have already taken it, please do not fill it out again. To make the text larger, use the buttons in the top right corner of the screen. To have the survey read out loud to you, click the speaker button underneath each question.

Why are we doing this research and why are you asked to participate?
You are being asked to participate in this research study because you are an adult with neurofibromatosis type 1 (NF1).

NF1 is a common genetic condition that affects about 1 in 2,500-3,000 people. People with NF1 experience various symptoms that can contribute to increased stress compared to the general population. Although there is significant research exploring stress in the overall NF1 population, little is known about whether or not a family history of NF1 impacts stress and coping.

The goal of this study is to investigate if a reported family history of NF1 influences perceived levels of stress and coping styles in adults with NF1.

If you decide to participate in this research, you will be asked to answer questions about your NF1 diagnosis and your family history, respond to questions about stress and coping, answer short response questions, and provide demographic information.

Do you have to be in this research and can you stop if you want to?
You do not have to be in this research study, and you can stop being in this study at any time. If you choose to stop being in the study, you can close the survey to discontinue and your responses will not be recorded.

What will you do and how long will it take?
If you agree to participate in our research study, you will take a one-time survey about your experience with NF1. It is estimated that this survey will take 30-45 minutes to complete.

Are there any risks or discomforts for this study?
This study will ask about your NF1 diagnosis and your experiences with stress and coping. These questions may bring up uncomfortable feelings. If you feel uncomfortable at any point, you can discontinue the survey. Below are some resources if this survey leads to feelings of discomfort:

NF Heartline: This is a hotline number designed to provide help and answer questions on your NF journey. Call the NF Heartline at 800-942-6825

NF Network: The NF Network offers resources about NF1 and ways to connect with support organizations. Visit their website at https://www.nfnetwork.org/

Children's Tumor Foundation: The Children's Tumor Foundation (CTF) offers resources about NF1 and ways to connect with other individuals affected by NF1. Visit their website at https://www.ctf.org/

What compensation will you receive for participating in the study?

You will receive a $10 Amazon e-gift card for completing this survey. This is a one-time payment that will be sent after the study ends. We expect you will receive your gift card no later than December 31st, 2023. To receive the gift card, you will need to provide your email address. Your email address will be collected via a second link at the end of the survey and will not be associated with your survey responses. The list of email addresses will be destroyed after gift cards have been distributed. Providing your email address is optional. However, you cannot receive compensation if an email is not provided.

How will your confidentiality and privacy be maintained?

The only identifying information we ask for is your email address (optional). After completion of the survey, you can select the link on the last page to be directed to a separate survey where you may provide your email address. This will keep your survey responses separate from your email address. This protects your confidentiality and data used by the research team for analysis does not include information that can be traced back to you. Your answers to the survey are linked only to a study ID number in a password protected electronic database. Only known study personnel will have access to the information. No identifying information will be included in any published findings. Electronic data will be kept indefinitely.

Who can you talk to about this study?

If you have any questions about this research study or possible risks, please feel free to contact me, Mikaela Bradley at mikaela.r.bradley@vanderbilt.edu, or my faculty advisor, Emily Solem at emilysolem@gmail.com

For additional information about your rights as a participant in this study, to discuss problems, concerns, and questions, or to offer input, please contact the Institutional Review Board Office at (615) 322-2918 or toll free at (866) 224-8273.

I have read the information sheet. All of my questions have been answered and I voluntarily choose to participate

○ Yes
○ No

Eligibility Questions: These questions are intended to make sure you meet the eligibility criteria to take our survey.

Have you been told by a doctor that you have Neurofibromatosis Type 1 (NF1)?	○ Yes<br>○ No
Are you 18 years old or older?	○ Yes<br>○ No
Do you live in the United States?	○ Yes<br>○ No
Can you read and understand English?	○ Yes<br>○ No

NF1 Questions: The following questions will help us better understand your personal medical experience with NF1.

How were you diagnosed with Neurofibromatosis Type 1 (NF1)? (Select all that apply)	☐ A doctor or another health provider<br>☐ I had genetic testing<br>☐ I have not been diagnosed with NF<br>☐ Other (Please elaborate below)
If you were diagnosed with NF1 in a way not listed above, please share more information:	__________________________________
At what age were you diagnosed with NF1?	◯ 0-2 years old (infant)<br>◯ 3-6 years old (early childhood)<br>◯ 7-12 years old (late childhood)<br>◯ 13-17 years old (adolescence)<br>◯ 18 years or older (adult)<br>◯ I do not know
Which of the following features of NF1 do you experience? Select all that apply.	☐ Café au lait spots (Flat, brown markings on the skin. Sometimes called birthmarks)<br>☐ Cutaneous neurofibromas (Small bumps on the skin)<br>☐ Freckling in your armpits or groin area<br>☐ Lisch nodules (Harmless yellow or brown flecks on the colored part of the eye that are seen on exam)<br>☐ Plexiform neurofibromas (Tumors that often involve branches of nerves. They can be on the skin or inside the body)<br>☐ Optic glioma (Tumors on the optic nerve. Optic gliomas may or may not lead to vision problems)<br>☐ Learning differences (Any difficulties with school or work related to areas such as learning, understanding, attention, or processing)<br>☐ Long bone dysplasia (The curving of a long bone, such as the tibia or fibula in the leg)<br>☐ Scoliosis (Curvature of the spine usually confirmed by x-rays)<br>☐ Malignant peripheral nerve sheath tumor - MPNST (A cancerous tumor that usually develops within plexiform neurofibromas)<br>☐ Seizures (Uncontrolled movements or epilepsy diagnosed by a doctor or by EEG)<br>☐ Osteoporosis (Decrease in bone mass and/or increased bone weakness)<br>☐ Hypertension (High blood pressure)<br>☐ I have additional NF1 features I would like to share
Please share any other NF1-related features you experience that were not listed above	__________________________________
How severe do you perceive your NF1 to be?	◯ Not severe<br>◯ Somewhat severe<br>◯ Moderately severe<br>◯ Very severe

**Family History Questions: The goal of our study is to assess if individuals' experiences with NF1-related stress and coping are impacted by their family history of NF1. Specifically, we want to know if individuals who also have a parent with NF1 experience stress and coping differently from those with unaffected parents. The next several questions will ask about your family history.**	
Has one of your biological parents been diagnosed with NF1?	◯ Yes<br>◯ No<br>◯ I was adopted<br>◯ I do not know
If yes, which of your biological parents has been diagnosed with NF1?	◯ Father<br>◯ Mother<br>◯ Both
If yes, in your opinion, how does the severity of your NF1 diagnosis compare with the severity of your parent's NF1?	◯ My NF1 is more severe<br>◯ My NF1 and my parent's NF1 are the same severi<br>◯ My parent's NF1 is more severe
If you were adopted, do you know if one of your biological parents was diagnosed with NF1?	◯ Yes, I know that one of my biological parents had NF1<br>◯ No, I do not know if one of my biological parents had NF1
Has anyone else in your family been diagnosed with NF1?	◯ Yes<br>◯ No
If yes, select all that apply.	☐ Aunts or Uncles<br>☐ Biological Children<br>☐ Cousins<br>☐ Grandparents<br>☐ Siblings (Brothers and Sisters)<br>☐ Other
How many aunts and uncles?	◯ 1<br>◯ 2<br>◯ 3<br>◯ 4<br>◯ 5+
How many biological children?	◯ 1<br>◯ 2<br>◯ 3<br>◯ 4<br>◯ 5+
How many cousins?	◯ 1<br>◯ 2<br>◯ 3<br>◯ 4<br>◯ 5+

How many grandparents?	◯ 1<br>◯ 2<br>◯ 3<br>◯ 4<br>◯ 5+

How many siblings (brothers and sisters)?	◯ 1<br>◯ 2<br>◯ 3<br>◯ 4<br>◯ 5+
How many other relatives?	◯ 1<br>◯ 2<br>◯ 3<br>◯ 4<br>◯ 5+
Total Other Relatives	________________________________

PSS-10: The perceived stress scale 10-item version is a self-assessment tool that helps us understand how different situations affect our feelings and our perceived stress. Stress can be defined as a state of worry caused by a difficult situation.

The questions in this scale ask about your feelings and thoughts during the last month. In each case, you will be asked to indicate how often you felt or thought a certain way.

To make the text and buttons larger, use the buttons in the top right corner of the screen.

	never	almost never	sometimes	fairly often	very often
In the last month, how often have you been upset because of something that happened unexpectedly?	○	○	○	○	○
In the last month, how often have you felt that you were unable to control the important things in your life?	○	○	○	○	○
In the last month, how often have you felt nervous and stressed?	○	○	○	○	○
In the last month, how often have you felt confident about your ability to handle your personal problems?	○	○	○	○	○
In the last month, how often have you felt that things were going your way?	○	○	○	○	○
In the last month, how often have you found that you could not cope with all the things that you had to do?	○	○	○	○	○
In the last month, how often have you been able to control irritations in your life?	○	○	○	○	○
In the last month, how often have you felt that you were on top of things?	○	○	○	○	○
In the last month, how often have you been angered because of things that happened that were outside of your control?	○	○	○	○	○

In the last month, how often have you felt difficulties were piling up so high that you could not overcome them?	○	○	○	○	○

Brief - COPE: The Brief-COPE is a self-report questionnaire designed to measure effective and ineffective ways to cope with a stressful life event. Coping is broadly defined as an effort used to minimize distress associated with negative life experiences.

When answering these questions, think about them in the context of your NF1 diagnosis.

To make the text and buttons larger, use the buttons in the top right corner of the screen.

	I haven't been doing this at all	A little bit	A medium amount	I've been doing this a lot
I've been turning to work or other activities to take my mind off things	○	○	○	○
I've been concentrating my efforts on doing something about the situation I'm in	○	○	○	○
I've been saying to myself "this isn't real"	○	○	○	○
I've been using alcohol or other drugs to make myself feel better	○	○	○	○
I've been getting emotional support from others	○	○	○	○
I've been giving up trying to deal with it	○	○	○	○
I've been taking action to try to make the situation better	○	○	○	○
I've been refusing to believe that it has happened	○	○	○	○
I've been saying things to let my unpleasant feelings escape	○	○	○	○
I've been getting help and advice from other people	○	○	○	○
I've been using alcohol or other drugs to help me get through it	○	○	○	○
I've been trying to see it in a different light, to make it seem more positive	○	○	○	○
I've been criticizing myself	○	○	○	○
I've been trying to come up with a strategy about what to do	○	○	○	○
I've been getting comfort and understanding from someone	○	○	○	○
I've been giving up the attempt to cope	○	○	○	○

I've been looking for something good in what is happening	○	○	○	○
I've been making jokes about it	○	○	○	○
I've been doing something to think about it less, such as going to movies, watching TV, reading, daydreaming, sleeping, or shopping	○	○	○	○
I've been accepting the reality of the fact that it has happened	○	○	○	○
I've been expressing my negative feelings	○	○	○	○
I've been trying to find comfort in my religion or spiritual beliefs	○	○	○	○
I've been trying to get advice or help from other people about what to do	○	○	○	○
I've been learning to live with it	○	○	○	○
I've been thinking hard about what steps to take	○	○	○	○
I've been blaming myself for things that happened	○	○	○	○
I've been praying or meditating	○	○	○	○
I've been making fun of the situation	○	○	○	○

Short Answer Questions: The questions in this section are intended to help us gain a better picture of how NF1 impacts your life.

What part(s) of your NF1 diagnosis is/are most stressful for you? ________________________________

What strategies do you use to cope with stressors related to your NF1 diagnosis? ________________________________

How did your parents talk about your NF1 diagnosis with you? ________________________________

Please add any additional comments you would like to share related to this study. ________________________________

Demographic Questions: We are collecting demographic information in order to report on the diversity of our study participants.

What is your current age? (Select from drop down menu)

What is your sex assigned at birth?	◯ Female<br>◯ Male<br>◯ Intersex<br>◯ Prefer not to say
What is your current gender identity? (Select all that apply)	☐ Cisgender Man<br>☐ Cisgender Woman<br>☐ Gender Non-conforming<br>☐ Non-binary<br>☐ Transgender Man<br>☐ Transgender Woman<br>☐ Two-Spirit<br>☐ Prefer not to say<br>☐ Add my own answer<br>(Gender identity may not align with sex assigned at birth.)
What is your current gender identity?	________________________________
What is the highest level of education you have completed?	◯ Less than a high school degree<br>◯ High school degree or equivalent (e.g., GED)<br>◯ Some college, no degree<br>◯ Trade or Tech School<br>◯ Associate Degree<br>◯ Bachelor's Degree<br>◯ Master's Degree<br>◯ Doctorate Degree<br>◯ Prefer not to say<br>◯ Other
What race(s) do you identify with? (Select all that apply)	☐ American Indian or Alaska Native or Native American<br>☐ Asian or Asian American<br>☐ Black or African American<br>☐ Middle Eastern or North African<br>☐ Native Hawaiian or Other Pacific Islander<br>☐ White or Caucasian<br>☐ Prefer not to say<br>☐ Add my own answer
What race(s) do you identify with?	________________________________
What ethnicity do you identify with? (Select all that apply)	◯ Hispanic or Latino/a<br>◯ Not Hispanic or Latino/a<br>◯ Prefer not to say

What is your current employment status? (Select the best answer)	◯ Full Time Employment<br>◯ Part Time Employment<br>◯ Not currently employed<br>◯ Disability<br>◯ Retired<br>◯ Student<br>◯ Other<br>◯ Prefer not to say
How did you hear about this survey?	________________________________

After clicking 'Submit', you will have the option to follow a link to provide your email address for compensation if desired.
